# Sir Alex. Crichton on Pulmonary Consumption

**Published:** 1823-06-01

**Authors:** 


*Practical Observations on the Treatment and Cure of several Varieties of Pulmonary Consumption; and on the Effects of the Vapour of Boiling Tar in that Disease.* By Sir Alexander Crichton, M.D. F.R.S. &c. &c. 8vo. London, December, 1822.

There is but too much reason to believe that the mode of applying the vapour of tar in pulmonary consumption, as recommended by our respectable countryman, Sir A. Crichton, has been completely mistaken in this kingdom, and consequently that the remedy has had no fair trial. On the contrary, we have no doubt that it has been productive of injury, as we shall shew a little farther on. On this account, and because the work before us contains some judicious observations on pulmonary consumption, independently of the remedy in question, we shall take a somewhat more comprehensive review of the publication than we otherwise should have been inclined to do, hoping that we shall not quite uselessly take up our readers' or our own time in the operation. The analysis of Sir Alexander's work is rendered very difficult by the want of arrangement and method which very generally pervades his chapters, which are composed in a desultory manner and loose style that we did not expect from an author who is well known to be capable of a very different kind of composition if he pleased.

In the year 1816, our author, while in Rnssia, made some experiments with the vapour of boiling tar in cases of consumption, and the succeeding year published an account of them in England, through the medium of a small pamphlet. Since that period he has employed the same means in private practice with success, "but not in the same degree as in the hospital for the poor in Russia." It is principally with the view of explaining the cause of this difference between the results of public and private practice that the present publication has been laid before his professional brethren.

After remarking, in his preface, on the incongruity of writers on pulmonary consumption, he considers it a well ascertained fact that the disease in question "cannot be cured by medicines which act through the medium of the stomach."

"It seems," says he, "a strange hope, and strange conduct, to pretend to cure an ulcer in the lungs, whether scrophulous, phlegmonous, or of whatever kind it be, by internal remedies alone, while it is acknowledged that ulcers on other parts of the body *require* a local application, independently of all internal treatment." xv.

We have put the word "*require*" in italics, because we are surprized that our author should suppose external applications absolutely *requisite* for the healing of external ulcers. On the contrary, we verily believe that these applications have very little other effect than to exclude the air and other irritating matters, and that the healing process is entirely the work of Nature, far more promoted by internal than external means. The analogy, therefore, between an ulcer on the leg and an ulcer in the lungs, if it holds good at all, will be directly against his principle of external applications.

We are ready to grant that there are well authenticated instances on record of pulmonary ulcers healing; but we are not equally ready to accord that—

"Almost all the gasses and volatilized substances which have been breathed by consumptive patients, oxigen gas and the nitrous oxide excepted, can boast of more cases of extraordinary cure, than have been effected by any medicine taken into the stomach."—*Pref.* xviii.

It is true they may "*boast;*" but are these boasts performed? We believe, with Sir Alexander, that tar vapour has been injudiciously applied in England, from imperfect acquaintance with his views and principles. For instance, when Dr. Lazaretto's pamphlet was published a few years ago, the pitch-house in Portmouth Dock-yard, where great cauldrons of tar were boiling, were daily crowded with patients labouring under pulmonary consumption, in every stage; as many as possible standing almost over these cauldrons, and all getting as close as possible to the supposed panacea. Numbers of them were forced, every minute, to run out into the cold air, and there cough and expectorate till they were red in the face, and until blood frequently appeared in the sputa. They then returned to their lodgings, many of them scarcely able to crawl, but all of them assuring the doctor how much they were bettered by this process! Many were the reports of surprising cures; but not a single such case did we see, although we were on the spot, and several of our own patients resorted, as long as they were able, to Medea's cauldrons in the dock-yard. We do not, in fact, believe that a single case of pulmonary consumption was cured, though not less than some thousands made the experiment. The concourse, at length, from all parts of the surrounding country, and even from a great distance, was so great, that the gates were shut—hundreds were cruelly disappointed—private lodgings were taken, and tar-pots smoaked for a few months, when the bubble burst, and the fame of the doctor, as well as the fume of the tar "vanished into air—thin air." Under such circumstance, we think, Sir Alexander Crichton has little reason to complain that his name was not coupled with the discovery; on the contrary, he may be really thankful, "as it absolves him (as he says, in another place, in mentioning the mode recommended in the Journal of Science,) from the charge of having advised so hurtful a mode of applying the remedy." It is but justice to our author to state, that the mode in which he administers the vapour is very different from those mentioned above; and also that he specifically informs his readers that the vapour of boiling tar is to be considered only as an auxiliary to the general treatment which the varieties of the disorder, and the various constitutions of individuals require.

In the first chapter of the work, our author appeals to his brethren respecting the *possibility* of curing some of the worst varieties of pulmonary consumption. The dissections of Laennec are adduced as proofs that tubercles have broken down, discharged their contents by expectoration, the cysts from which the discharge came having healed by a kind of cicatrization.* These facts, and the histories of cases which have fallen under our author's notice, or been recorded by others, authorize, he thinks, the hope that something may yet be done to lessen the fatality of pulmonary consumption. The following statement, however, will shew that the tar vapour is not likely to be extensively tried in this country with advantage, under present circumstances.

"No fair trial of its virtues can be made indeed in this country, in any of its establishments or houses, as at present constructed; a circumstance, of the importance of which I was not fully aware, when I published an account of the first trials made with this remedy; for having been accustomed, during a residence of sixteen years in Russia, to the uniform temperature of the houses and hos-

---

* See the 7th number of our *Quarterly Series*, for January 1820, p. 467 et seq. Also, Forbes' Translation of Laennec, p. 25.

pitals of that country, I was not sufficiently aware how much this circumstance favored the operation of all other means employed for the recovery of the consumptive."* P. 7.

Sir Alexander is convinced, however, that temperature alone is incapable of effecting a cure, when the disease is fairly formed, as is proved, he thinks, by the cases treated in 1816, where the patients had all the advantages of uniform temperature, and were daily getting worse, until they were made to breathe an air charged with the vapour of heated tar.

Where the substance of the lungs is much disorganized by the presence of a great number of tubercles, whether dormant or in a state of progression, our author acknowledges that no man in his senses can flatter himself with the hope of a cure. Nevertheless, experience has proved that life may continue after a very considerable portion of lung has ceased to perform its function; and, therefore, the hope of recovery is not absolutely abolished in such cases. The following is the supposed modus agendi of tar vapour.

"The vapour of boiling tar seems to act in different ways on tubercular and ulcerated lungs: first, as a stimulus both to the arterial and absorbent system of the diseased parts, *hastening the softening or resolution of tubercles*, and the generation of new matter; the remedy coming into immediate contact with the diseased parts: and secondly, by diminishing the quantity of oxygen in any given mass of air, not by any chemical union with it, although this remains to be proved, but, as I suspect, merely by diluting the atmospheric air with a vapour which contains little or no oxygen, and is almost entirely composed of carbon and hydrogen, and thereby lessening the quantity of that æriform fluid which seems to augment the suppurative process of all sores." 14.

Pathologists will differ as well as *doctors* some times. Our readers will remember that Dr. Baron, who has paid no mean degree of attention to the subject under consideration, founds one of his greatest hopes of cure on the *hardening of the tubercles*, and their *thus* lying in a dormant state through life. Sir Alexander seems to think that the tar vapour *softens* tubercles, and promotes the *generation of new matter*.

Our author informs us that the success of the tar vapour has been quite remarkable in some cases, while in others,

* As it may be in the power of many opulent individuals to command the means of regulating the temperature of their apartments, and thus availing themselves of the tar fumigation, we still deem it our duty to lay before the profession an analysis of the work before us, notwithstanding the above draw-back on our prospects.

apparently similar, it did no good—in others still it evidently provoked hæmoptoe.

"In almost all cases where the tar has been duly prepared, its first effects in relieving the dyspnœa, cough, and pains in the chest, and in diminishing the expectoration, have been remarkable. These effects, on such patients, as from the state of the lungs were curable, were soon followed by a progressive and general amendment until a complete cure was produced. But others, who at first were much benefited by the application, began some time after to struggle, as it were, between the good effects of the medicine, and the destructive process of the disease, and from the number and volume of the tubercles, they at last resisted its action altogether." 15.

That tubercles occasionally become absorbed, our author thinks is sufficiently probable from some cases on record, and among others Dr. Young's own case. The vapour of tar, he observes, if long continued, of moderate force, and uniform temperature, has seemed to him to produce this happy change. Hence, he recommends it to be tried in the dry or tubercular consumption anterior to purulent expectoration—but with this caution, for fear of hæmoptoe, "that the air of the apartment be charged with a due degree of humidity as well as of the vapour of tar."

"The most destructive variety of consumption, and one of the most frequent, is that arising from the rapid succession of *inflammation and suppuration of many clusters of tubercles.* It is distinguished by the frequency of the cough, a rapid decay of strength, pains in the chest, and a constantly quick pulse, which is generally wiry and hard, until the weakness becomes general.

"To the well informed part of the medical profession it would be superfluous to attempt a picture of the morbid appearances of the lungs in the various stages of this disease, after what has been already written on the subject by Messieurs Bayle and Laennec. But, in a practical point of view, all that is necessary to state is, that as long as a quick and rather hard pulse accompanies the other usual symptoms of tubercular phthisis, it may be relied on that a scrophulous inflammation of an extensive and disorganizing kind is rapidly going forward, and in such cases the vapour of tar, although it now and then relieves the frequency of the cough, produces only a temporary amendment, but cannot cure the disease until the scrophulous action is arrested, which, in such cases, is almost hopeless in the greater number of similar cases." 21.

Our author also remarks, that "as by far the greater number of consumptive cases which occur in Great Britain are of this acute and destructive kind, the vapour of tar, should it have been solely trusted to, must have failed; for such cases, even when a little hope is to be entertained, demand the co-operation of internal remedies."

Now we humbly conceive that, if we can, by internal remedies, check this scrophulous "inflammation and suppuration of many clusters of tubercles," the patient will be saved without tar vapour—that is, that the remedies which can effect such important objects as the above, will do the remainder of the work better than the stimulus* of tar vapour, on parts but too much disposed already to inflammatory action.

The quick and hard pulse, with strongly marked hectic fever, which accompanies all the varieties of pulmonary consumption arising from extensive tubercular inflammation, are infinitely less frequent, our author observes, among the poorer inhabitants of Russia than of Great Britain or France; and hence, together with the construction of their habitations, the greater success attending the administration of tar vapour in the former class of patients.

Dr. Neuman, physician to the Charité at Berlin, assured our author that he had succeeded in completely curing, by means of tar vapour, that dangerous disease known by the name of laryngeal phthisis. In the autumn before last, Dr. N. shewed our author a case of this kind, which was gradually recovering by means of the tar vapour. We should indeed have more expectations from the remedy in laryngeal, tracheal, and bronchial ulcerations, than in real tubercular consumption, and for a reason which, we think, has escaped Sir Alexander. We firmly believe, that atmospheric air rarely, if ever, enters the cavity of a broken-down, or, as it is commonly called, suppurated tubercle. During the intervals of coughing, or at least of expectoration, the cavity is gradually accumulating muco-purulent matter, which, of course, excludes the atmospheric air; and when a fit of coughing and expuition takes place, so as more or less to clear the excavation of its morbid contents, the parietes of that excavation are in contact, or nearly so, (for they are never completely emptied of their contents,) till they become gradually distended with the muco-purulent secretion, and again excite the paroxysm of coughing and expectoration. We imagine that every physiologist who reflects on the mechanism of the lungs and the phenomena of expectoration and consumption, will agree with us in this view of the case. No man, we think, can coolly believe that a tubercular cavity, whose parietes are in a secreting state, are dilated with air at each inspiration, and collapsed at each expiration. The idea is quite

---

* In an extract which we quoted, our author considers the tar vapour "a stimulus both to the arterial and absorbent system"

preposterous. And if we are right in our exposition of the case, tar vapour can rarely, if ever, permeate the cavity (as it is erroneously called, for there is no real cavity*) of a suppurated tubercle, and, consequently, whatever effect it may produce, must be accounted for on other principles than those attending applications to external ulcers.

Our author observes that the tar vapour has been successful in bronchial phthisis, and we think it very probable—especially in Russia, where a uniformity of temperature is easily commanded in the apartment of the sick. The reason why it has not been so efficacious in this country, may, as our author states, be much owing to the want of power in regulating the temperature of our houses in England. Our author properly observes that the inhalation of vapour from a gazometer or bag must be injurious in consumptive cases, not only on account of the exertion required by the chest, but of the too stimulating quality of the inhaled vapour. Whatever this latter may be, it is important that the whole chamber should be equally impregnated therewith, in order that the patient may breathe without any greater exertion than usual. In fact, our author considers it desirable that the consumptive person should take in as little air as possible into his lungs, and, consequently, that there should be as little motion as possible in the respiratory apparatus.

The places where the good effects of tar vapour become most conspicuous, are the cable-lofts in the neighbourhood of pitch-houses, when great quantities of tar are boiled, and impregnate the air to a considerable distance around. But our author *distinctly states that it is not in the chamber (of the manufactors) where the tar is boiled, but at a considerable distance from it in the building, that a consumptive patient can breathe the air with safety.* This at once shews that the trials to which we alluded at Portsmouth must have done more harm than good.

"The object is to find a place where the atmosphere is highly charged with the vapour of tar alone, the pyroligneous acid having been dissipated before the vapour reaches the spot alluded to. This acid has a very sensible effect on the eyes, and this circumstance is a good practical criterion for judging of its presence. The common tests for discovering the presence of an acid are no tests whatever in this case, because they are quickly reddened by the carbonic acid gas, which abounds in such manufactories, and which does no harm." 29.

* The cavity of a dischaging tubercle may be compared to the cavity of the abdomen in a dropsical patient, who is in the habit of being tapped. It alternately fills and is emptied, but there never is any cavity, properly speaking, in either state.

In the manufactories alluded to, the patient should remain, weather permitting, as long as he can bear it, viz. until giddiness or violent head-ache forces him to retire; and if he is fortunately in the vicinity of such an establishment, he ought to repeat this visit several times a day.

"The success of the cure depends very much on the impregnation of the air with the finer and more permanently volatile parts of the tar, an advantage which it is scarcely possible to procure by the boiling of small quantities of tar over a lamp or charcoal furnace in the apartments of the sick, except this be done with great skill, attention, and patience. But, if the sending consumptive patients twice or thrice a day to some manufactory where tar is boiled in great quantities be advantageous, it is only so in mild weather; for the pernicious effects of a cold or windy day, to which the patient must occasionally be exposed who seeks the remedy out of his dwelling, will do more injury than can be counterbalanced by the pitch-house; and this forms another reason why establishments ought to be erected in various parts of Great Britain for this unfortunate class of sufferers, provided the trials which are first made in this manner affords a prospect of their utility; and it may be added, that if ever there was an interesting, useful, and praiseworthy object of experiment and of humanity, it would be the establishing houses for such trials in this country, where so much domestic misery arises from the prevalence of the disease." 31.

That a large or expensive establishment, however, is not always or essentially necessary, our author relates successful cases where the tar fumigation was made by keeping about a quart of tar in a state of slow ebullition, over a spirit lamp, in one corner of the patient's room.

But although our author saw many cases where patients recovered in an atmosphere constantly charged with the vapour of boiling tar, he by no means recommends the trusting to this method, in preference to the interrupted and repeated action of an atmosphere more highly charged. Sometimes the one and sometimes the other method will agree best.

The second chapter of the work before us is on the influence of temperature and climate in preventing and curing consumption. Sir Alexander regrets that patients are still sent to Nice, Italy, France, Naples, and Madeira, "all which places are destructive to these unfortunate people." Surely this is an unguarded and unqualified expression. That patients are sometimes sent to these places at improper periods of their complaints, and at unseasonable times of the year, there can be no doubt; but this affords no proof surely that a judicious and timely removal to a milder climate is "destructive" to those who go under such restrictions.

Consumption is infinitely more prevalent in Great Britain than in the northern parts of Russia, although the climate of the latter is far colder and ruder than ours. In the *northern and middle* governments of Russia, however, the scrophulous constitution is more common than in England, and commits greater ravages and disfigurations than are ever seen here. In that country the disease is principally confined to the external glands, especially to the face, eyes, throat, and bones. The lungs rarely suffer, except in public schools, and among those who adopt the European dress and fashions.

The chief food of the Russian peasant is a black sourish rye bread, (less nutritive than that of wheat,) and a very weak cabbage soup. Almost all his drink is acidulated and fermented. During his long religious fasts he is obliged to eat black bread, bad hemp-oil, and vegetables, to the total exclusion of every article of diet derived from animals, whether flesh or fish. Even milk is strictly prohibited. It must be confessed that this does not seem to be a diet calculated to either prevent or cure a scrophulous diathesis—but, at the same time, it must deprive the lungs of all tendency to inflammatory condition. The peasantry are warmly clothed, except during the excessive heats of their short summers—their huts being insupportably hot during winter, spring, and autumn, in comparison with our houses. High and frequent winds, a fruitful source of catarrhal affections in this country, are very rare in the northern parts of Russia; and when they do occur, every Russian who has it in his power shuts himself up.

"And as the peasantry of both sexes are clothed in sheep skins, with the fur turned inwards, they are not quickly robbed of their atmosphere of heat on leaving their huts, and consequently can bear almost any transition of temperature with impunity. The lungs alone are exposed to the cold; but the great mass of blood is not thrown on these viscera by sudden exposure to it, as it is in this country; and the circulation being kept up freely on the surface of the body, and in the extremities, no injury arises." 52.

The higher classes, however, who are now well inured to the tyranny of European dress and manners, are daily becoming more and more subject to phthisis. What has been said of the Russian peasantry appears to apply to those of Sweden and Lapland, where, according to authentic travellers, the disease in question is very rare.

"These facts, if well considered, lead to this conclusion; that the best preservative against pulmonary complaints is warmth, and an equal, or at least an uniform mild temperature, so as to keep the balance of blood constantly on the surface." 53.

Our author observes that the more *steady* climates of France,

Spain, and Italy, must be the chief cause why consumption is less common in those countries than in Great Britain.

"None of these countries are altogether free from vicissitudes of temperature, and those to which consumptive patients are commonly sent from this country are exposed, during hot seasons, to sudden cold and cutting winds, from the glaciers and high mountains in their neighbourhood, which alternating with siroccos and hot winds, soon cut short the days of a consumptive patient. The whole of Dauphiné, almost the whole of the South of France, the North of Italy, and indeed all places which are under the influence of cold winds from the Alps, Appenines, or Pyrenees, are subject to phthisis, and destructive to foreigners labouring under that complaint, or who are disposed to it from diseased and weak lungs." 54.

Sir Alexander asserts that the sea coast, whatever may be the locality, is highly injurious to phthisical patients, provided there be any breach of continuity in the pulmonary apparatus. Where the ulceration is not present, these maritime situations are allowed to be salutary. But our author shews no good physiological or pathological reason for this injurious influence in one case, and beneficial to the other; and as a matter of mere experience, he shews no proof at all. Neither is our author's statement consistent with the very next passage in sequence.

"Some places on the coasts of Devonshire and Cornwall, such as Penzance, have been long considered as favourable to the recovery of the consumptive. If the fact be well founded, which I am much inclined to believe it is, the reason is to be sought for in the steady, mild, and perhaps humid state of the atmosphere of these favoured spots, but most assuredly not in any specific virtue of the sea air." 57.

No man of sense or reflection now entertains any idea of a "*specific virtue*" in the air of particular places; and, therefore, Sir Alexander here combats a mere man of straw set up by himself. Madeira is considered improper for those in whom phthisis is fully formed—but salubrious for those in whom the disposition only exists, or the tubercles are merely nascent. Very hot climates, he observes, are "quite free" from consumption, "such, for instance, as Persia, and *many parts of Calcutta*." We have traversed over and over every nook and corner of the last-mentioned city, without ever hearing of these favoured spots. Although phthisis is comparatively rare in the hotter climates of the earth, there is no such exemption, we believe, in any of them, as our author points out.

Sir Alexander does not believe that marshy countries possess any anti-phthisical properties, unless they are much sheltered from cold winds:—

"And provided the air of such places be loaded with moisture alone, or only with a little carburated hydrogen, I can imagine that they may be favourable to the consumptive. The effluvia and volatilized substances, arising from the decomposition of animal matters, is always useful to people of weak lungs, and to the consumptive. Cat-gut-makers, who live in a putrid atmosphere, and butchers, are of all tradesmen those who are the least frequently affected with this disease." 59.

Our author's observations on the effects of sea voyages in consumption, correspond with our own. Where tubercles are discharging, a voyage to a hot climate generally accelerates the progress of the disease. But the mild and comparatively equable temperature of the ocean is beneficial for those in whom there is but a tendency to phthisis or hæmoptysis. In chronic bronchitis, even where there is actual secretion of muco-purulent matter, the atmosphere of the ocean is also salutary for the reasons abovementioned—namely, its *stability and mildness*. The chapter concludes thus:—

"The view which has been taken of the influence of climate and temperature, shows on the one hand how very little is to be expected from either topical applications to the ulcerated lungs of consumptive patients in Great Britain, or from internal remedies, if some plan be not adopted to shelter the patient, during his recovery, from the frequent vicissitudes of temperature to which we are subject; and, on the other hand, the cases brought forward justify the hope, that with the advantages of an artificial and equal temperature, and due regard to diet, exercise, and internal means, the efficacy of the vapour of boiling tar on ulcerated lungs, might rescue many lives which must otherwise be lost." 68.

The third chapter of the work is on tubercular consumption, its prevention and treatment. There is nothing new or original in the pathological part of this chapter. Our author quotes good authorities, however, as Baillie, Bayle, and Lænnec. He has omitted Dr. Baron, whose work on tuberculated accretions he would have done well to peruse. Neither do we see much new or original matter in the hygiene contained in this chapter. Our author's observations are very generally correct, and his advice proper; but to us it seems more intended for the patient than for the physician. On this account we shall take but a very cursory view of the chapter.

Our author divides those who are unfortunately born with "a strong hereditary disposition to scrofula" into two classes —one, where the child is fair and well proportioned, until it begins to be weakened by growth, and by those debilitating causes which spring equally from wealth and indigence —the other, where the child is born with a delicate frame, and is soon observed to have a flat and compressed chest, transparent skin, long neck, and other well known indications of weakness which, with hereditary taint, mark them out as easy victims to pulmonary consumption. Although the first class may have a well-formed skeleton, plenty of flesh and blood, and a healthy colour, they may yet have a tendency to vascular derangement in one or more parts of their system, disposing them to glandular or membranous inflammation. Still these subjects seldom fall into actual phthisis, unless the external causes be in considerable force, with neglect on the part of themselves or others.

Our author descants on the improper conduct of some parents in attempting to harden their offspring by early inuring, or rather exposing them to cold, and the natural inclemencies of the climate. We confess that we can only see the impropriety of this measure when it is carried to extremes. We know not how the human frame can be rendered capable of sustaining the rapid and frequent changes of our atmosphere but *by early and gradual exposure* to those changes. We would not indeed begin by plunging a tender infant into water half-frozen; but we would caution mothers against bringing up their children like hot-house plants, that cannot bear the breath of heaven without shrinking from the exposure. We would recommend *moderately* warm clothing and plenty of exercise in the open air, with the cold bath in summer, as the surest means of hardening the constitution. Our author properly recommends that a scrophulous child should be in the open air several times a day, and that he should have wholesome animal food, unless when labouring under some actual disease. Sir Alexander has also given some judicious instructions respecting the exhibition of mercurial alteratives in scrophulous diseases. About the age of puberty the scrophulous diathesis too often assumes the form of tubercular phthisis, and therefore it is important to watch the first movements that indicate danger to the lungs.

"Where a little dyspnœa and slight frequent cough have already commenced, an uniformly mild temperature, breathing an atmosphere charged with the vapour of heated tar, as a topical stimulant to the absorbents of the lungs, sponging the chest frequently with tepid vinegar and brandy, in an apartment sufficiently warm to prevent the catching cold, rubifacients, the tartar emetic ointment, a mild yet nutritious diet, and keeping the bowels gently open, are the best remedies." 86.

The circumstances indicative of the formation of tubercles in the lungs, our author observes, are the rapid emaciation and loss of strength, although a sufficient quantity of food is daily taken in—a frequent dry cough—occasional dyspnœa on taking exercise—flying pains in the chest—or a fixed and moderate pain in either or both sides—a peculiar shrinking of the features, and delicacy of look.

Sir Alexander maintains that there are two distinct classes of tubercular consumption—one of slow and insidious progress, accompanied by all the decided marks of phthisis, except a quick pulse, and daily symptomatic fever—the other characterized by hectic fever, with daily exacerbations. "These are not stages of one and the same disease;" but dependent on the constitution of the patient, and the state of the lungs. The first, which he thinks may be called the chronic kind, though ultimately accompanied by hectic fever, may yet run a course of one, two, or even several years, before the hectic fever occurs. Whereas the acute kind is accompanied with quick pulse, and daily exacerbations of fever from almost the beginning of the complaint, running, in comparison with the other, a very rapid course.

"In the chronic kind, the formation of fresh tubercles goes on slowly, and the enlargement of each tubercular mass, and its progress in softening, is also slow and unaccompanied by inflammation. When one bag of softened tubercular matter has been evacuated by coughing, the patient apparently recovers a little, the frequency of the cough is sensibly diminished, and there is a general improvement in his health for a short time; he has a kind of fallacious respite, which is often attributed to the operation of medicine, which, if judiciously applied, certainly does assist Nature in her operations. In a certain lapse of days, weeks, or even months, the dyspnœa increases, the patient has bad nights, his strength fails, and at last there is a fresh attack of cough, with the mixed expectoration peculiar to tubercular consumption. Of those who recover, I have known some who have struggled in this manner, for nearly a year before they could be said to be in a state of uniform convalescence, and consequently before the greater number of tubercular masses were resolved. In women, the non-appearance of the catamenia, which is always a concomitant of an advanced phthisis, does not occur in this chronic kind until the last periods of emaciation, whereas, in the acute kind, it generally occurs soon, the *rationale* of which is too evident to require explanation." 89.

Although the prognosis is here unfavourable, our author thinks that a tenth part of those attacked might be saved, if the disease were taken in time.

"People labouring under this variety of consumption, as well indeed as all others who suffer from the other varieties of the disease, ought to be confined rigidly to the house during winter, and almost equally so during summer, except the weather be very calm and warm; in which case I allow them to take as much exercise as their strength permits, twice or even thrice a day. The apartment is always kept charged with the vapour of tar, duly prepared, sometimes more strongly charged than at others, while the dryness of the air is obviated as much as possible. All tea and coffee and acid drinks are forbidden.

"If the patient has sufficient appetite to relish good soup of beef or mutton for breakfast, I prefer this to every thing else; if not, he has cocoa, milk, or chocolate, according to the state of his stomach, and according to the facility with which he digests them.

"If he have a craving for food, or much appetite, calf's-foot jelly is permitted between breakfast and dinner, as often as the patient wishes for it. At dinner he has any variety of animal food which agrees with him, but he must confine himself to one kind each day. He is allowed no *entremets*. He has, in fact, soup and bouillie, or soup and some roasted or broiled meat in moderate quantity." 91.

The medicines which our author has found most effectual in promoting the resolution and discharge of the tubercular matter, have been a combination of the compound galbanum pill with the sulphurated oxide of antimony, or the pulvis antimonialis—now and then calomel or cinnabar, and always some extract of hemlock and a little opium. We agree with our author "that very many compound medicines answer better than simple prescriptions."

"To those who think that the science of medicine is improved by an affected simplicity in prescribing, I would remark, that modern pharmacopœias are shorn so much of old and approved receipts, on account of their being extraordinary compounds, as to be almost quite useless in some cases." 92.

Along with the above-mentioned pills, our aurthor gives the Iceland moss and sarsaparilla, particular symptoms being obviated by appropriate remedies.

The inflammatory species of consumption above alluded to, must be treated in a different manner—namely by "remedies which experience has pointed out as the most efficacious in allaying febrile action, the scrophulous affection and the frequency of the cough naturally modifying the prescription."

"These remedies, considered under a general point of view, are such as act on the skin, and consequently tend to unload the lungs of the habitual congestion, to which their weakness, and the irritation arising from tubercles, give birth; and, secondly, mild stimuli, the action of which is directed to the lungs; and, thirdly, mild general tonics. In one word, the cure must be anti-febrile, and anti-scrophulous, and not merely anti-catarrhal, as it is but too often attempted to be." 100.

Antimony, digitalis, cicuta, and the red sulphuret of quicksilver, are the principal remedies to which our author trusts for allaying febrile action; to which must be added, as external counter-irritants, blisters, tartar emetic ointment, gestation. Finally, regulated temperature and the tar vapour, when inflammatory symptoms have subsided. Griffith's mixture our author condemns; but "the watery extract of myrrh is an excellent tonic." The Iceland moss is also recommended. Of milk and other diatetic articles we need not here speak.

The 4th chapter (which our author has marked the 3d) is on pulmonary consumption from hæmoptysis. The pathology is almost entirely taken from Laennec, and although there is some peculiarities in the therapeutics of our author, we do not deem them of much importance. However as he seems to be of a different opinion, we shall state his practice in a very few words, leaving our readers to form their own opinions.

He condemns the application of cold water to the face, temples, hands, &c. of a patient labouring under hæmorrhage from the lungs. He advises the air of the apartment to be cold, but the feet and legs to be immersed in warm water, or wrapped up in warm woollen dress, the head, neck, and chest being left exposed. As soon as the circulation is free in the extremities, the patient is to be freely blooded, and next a blister is to be applied to the sternum and another to the back. Instead of giving alum, the mineral acids, and other astringents, he gives a combination of the nitrate of potash and sulphate of magnesia every two hours, until one or two loose stools follow, after which he trusts to emollient drink, with nitre and cream of tartar.

There are two principal objects to be kept in view, we apprehend, in hæmoptysis—first to arrest the hæmorrhage, and secondly to prevent the inflammation which usually follows. Cool air, blood-letting, and the superacetate of lead with opium, we have found the most powerful medicines and means in arresting the hæmorrhage, opening the bowels once a day. The inflammation which succeeds is to be treated on the same principles that guide us in inflammation of the lungs in all similar constitutions. We have not found phthisis so *very generally* succeed hæmoptysis as our author and most other writers would lead us to expect.

The 4th chapter is on consumption arising from neglected perepneumony. This disease is rare in Russia, the fur dresses obviating its causes—sudden transitions of temperature. Our author questions the correctness of Laennec's statement, that the termination of pneumonia in abscess or suppuration of the lungs is a comparatively rare event. With due submission to Sir Alexander, we attach more credit to the researches and observations of Laennec. The breaking down and discharge of tubercles has, times out of number, been mistaken for abscess in the parenchymatous structure of the lungs. This chapter offers nothing on which we can dwell.

The 5th chapter is on bronchitis ; but after having given a circumstantial account of the valuable work of Dr. Hastings, we cannot hope to present our readers with any thing new out of the present chapter.

The 6th chapter treats of the prevention of bronchitis, and consequent phthisis, from measles. Our author condemns the cold treatment in measles—a practice which is very little adopted, we believe, in England—we mean cold effusion, &c. and recommends the keeping up a very mild but constant moisture on the skin, especially during the whole of the eruption, and for a day or two after, by rigidly prohibiting all those drinks which are not tepid, whether simply aqueous or mucilaginous, and by keeping the patient in a regulated temperature. In severe cases, of course, we are to employ local and general bleeding, and blisters, in addition to warm air. In our experience, there has not been that great degree of gastric irritability described by our author, which forbids the use of antimonials, and especially of effervescing draughts. Our author's mode of treatment is, however, upon the whole judicious.

The 7th chapter is on laryngeal and tracheal phthisis. The former is acknowledged to be almost entirely hopeless, when once established ; but from some late trials our author has reason to hope it will cease to be so incurable.

"The only remedy which has hitherto arrested this fatal complaint has been the vapour of heated tar; and it promises to be effectual in most cases if taken in time, and if administered with all the caution which has been recommended. But the confining patients, labouring under this variety of phthisis, to an uniformly mild temperature, is also a most essential part of the cure. Patients labouring under this disease ought to speak as little as possible. The best internal medicines are the Iceland moss in large doses, and the balsam of copaiba, with the balsam of Tolu and sulphur at bedtime.—*See Formula* 28.

"This last combination of medicines, which it occurred to me to try only of late, produced very decidedly good effects in a case which fell under my care. Where there is much pain, blisters afford a temporary relief: opium and its preparations do harm." 210.

The 8th and 9th chapters are headed thus:—is consumption hereditary?—is consumption contagious?—It is quite unnecessary to say a word respecting these two subjects now-a-days in this country. We shall, however, offer an extract on the second question from our author.

"It cannot be asserted that a specific contagion is generated in the lungs of consumptive people, because the ulcers which exist in that disease, not being in every case of the same kind, they cannot be supposed to generate the same poison; but the result of my experience in regard to this subject is, that where the air of an apartment is strongly impregnated with the breath of any one, or any number of consumptive people, labouring under the advanced stages of any variety whatever of purulent phthisis, a poison is often produced by the chemical influence of temperature on a confined air thus loaded with vitiated animal effluvia, just as the poison of scarlatina is often generated from temperature acting on air loaded with the breath and perspirable matter of a number of children, when confined in small apartments.

"That a healthy person sleeping constantly with one who has an open ulcer in the lungs, and especially where the ulcerated surface is large, or where the ulcers are numerous, and where a vitiated pus is formed, almost infallibly gains the disorder, is a fact which has occurred to me to see so often, that I have no doubt on the subject. It has fallen repeatedly to my lot, particularly among the poor, to see a husband gain the disease from his wife, and many wives gain it from their husbands, under circumstances where there could be no doubt as to the influence of the poison, and no other way of accounting for the disease." 223.

The 10th chapter is on the method of employing the tar vapour. The best tar, for the purpose under consideration, is that which is used in the navy and in cable manufactories. As it comes to market it is generally found to be contaminated, more or less, with pyroligneous acid, which being very volatile, is disengaged long before the tar boils. On this account the tar ought to be boiled for a few minutes in the open air, and then one or two ounces of the subcarbonate of potash added to each pound of the tar. Some water should next be put to it, and the whole well stirred together. When a visible whitish vapour rises in boiling, there is too much heat, or there are impurities in the tar. These circumstances cause coughing and irritation in the patients. In private practice, two adjoining apartments ought to be dedicated to the vapour, by which means each can be easily ventilated when necessary. Our author causes the door on the stair-case to be closed, and the windows well secured. The simplest way of charging the apartment is to put about a pint of the prepared tar into any flat dish of iron, copper or earthenware, which is to be placed about a foot from the ground, with a suitable lamp under it. It is to be regulated by the patient's feelings, and the temperature of the room is to be kept as nearly to 60° as possible.

"From what has been stated, it must appear that the chief *desiderata*, in constructing any establishment for the consumptive in such a climate as ours, are few in number, and yet of great importance.

They may be reduced under the following heads:

"1st. The possibility of charging, to any degree of concentration, all or any of the apartments with the tar vapour.

"2d. A mode of ventilation which shall not expose the patient to a current of cold air during our cold seasons, or indeed at any time.

"3d. The possibility of having one large ward or room always fully charged, and of a due temperature, to which patients can resort for exercise.

"4th. The means of preserving an equal temperature in every part of the establishment to which the patients are admitted.

"All these essential objects may be attained by means of two chambers in the under part of the house; the one being a reservoir of heated air, the other a reservoir of the vapour. From these tubes of communication may be easily conveyed to the different parts of the establishment.

"The windows ought to be double, and two ventilators ought to be in each apartment: one in the centre of the roof, the other on a level with the floor. The first to allow the escape of superfluous heat, the second that of any heavy air. These are naturally to be opened only at times." 237.

Here we must close our analysis, having made our readers sufficiently acquainted with the nature and scope of Sir Alexander's work, to enable them to judge of its relative degree of importance. Considering that all the novel matter which it contains, might have been compressed into a very small pamphlet, or even a paper in a periodical journal, we cannot see the necessity there was for publishing a volume on the subject. If, however, the work never rises above, it never sinks below mediocrity, and therefore there is no demand made on the reviewer for either praise or censure.

